# The use of artificial intelligence and machine learning to predict tumor recurrence in high-grade gliomas: a systematic review

**DOI:** 10.1007/s10143-026-04406-7

**Published:** 2026-07-23

**Authors:** Trent Kite, Tushar Nayak, Stephen Jaffee, Mokshal Porwal, Cynthia Han, Praveer Vyas, Aswin Sankaranarayanan, Rodney E. Wegner, Pulkit Grover, Matthew J. Shepard

**Affiliations:** 1 Department of Neurosurgery, Allegheny Health Network Neuroscience Institute, 320 E North Ave, Pittsburgh, PA 15212 USA; 2 Department of Electrical and Computer Engineering, Carnegie Mellon University, Pittsburgh, PA USA; 3 Division of Radiation Oncology, Allegheny Health Network Cancer Institute, Pittsburgh, PA USA

**Keywords:** High-grade glioma, Artificial intelligence, Neural networks, Prediction, Machine learning

## Abstract

High-grade gliomas (HGGs) are aggressive tumors with a propensity for recurrence. Despite standardized therapies, definitive treatment is elusive. Advancements in artificial intelligence (AI) and machine learning (ML) can potentially identify recurrence probabilities and patterns facilitating individualized therapy. A systematic review was conducted in accordance with the PRISMA guidelines. PubMed, ScienceDirect, and Web of Science databases were queried for reports on the use of AI/ML for the prediction of disease recurrence in HGGs. Using a random-effects model and inverse variance weighting a pooled analysis of key performance metrics (sensitivity, specificity, and accuracy) was conducted on the top performing models from each manuscript. In total, 14 manuscripts encompassing 1,540 patients were selected for systematic review and analysis. Across the included studies, 13/14 (92.9%) were retrospective study designs, with 1/14 (7.1%) prospective study design. Among the 1,540 patients, 1,530 (99.3%) and 10 (0.7%) were histologically classified as WHO grade IV and III respectively. Nine studies (9/14, 64.3%) examined patients undergoing GTR following by adjuvant RT, and five studies (5/14, 35.7%) undergoing STR/NTR followed by adjuvant RT. The Random Forest (RF) model was most frequently utilized, with T2-FLAIR sequences most frequently incorporated into model training. The pooled sensitivity, specificity, and accuracy of the models were 81% (95% CI: 73–87; I² = 85.2%), 75% (95% CI: 65–85; I² = 91.9%), and 79% (95% CI: 64–92; I² = 87.8%), respectively. Following sensitivity analyses, the corresponding estimates were 81% (95% CI: 77–84; I² = 0.0%), 84% (95% CI: 79–88; I² = 38.8%), and 89% (95% CI: 83–95; I² = 0.0%), respectively. The development of an AI/ML model to predict tumor recurrence in HGGs has been an emerging area of research over the past decade. While ongoing validation in larger, prospective databases is needed, preliminary evidence suggests that existing models perform with reasonable sensitivity, specificity, and accuracy.

## Introduction

High-grade gliomas (HGGs) are aggressive primary intracranial tumors associated with a poor prognosis, despite standardized care [1]. The aggressive nature of these tumors mandates maximal safe cytoreductive surgery and adjuvant multimodal therapy with radiation and chemotherapy [2]. Despite this aggressive approach, the median overall survival (OS) remains at 12–16 months [1].

The diffuse nature of HGGs complicates the ability to achieve tumor control by surgical excision alone. It is well-documented that HGGs can have high subclinical tumor dissemination at the time of diagnosis, but determining the extent of the disease and predicting tumor recurrence is difficult. As a result, an effort to perform supramaximal resections from the treatment outset has been suggested to improve patient outcomes [3, 4]. Increasing the extent of resection beyond the enhancing tumor margin, while technically feasible in select cases, is not currently guided by an understanding of what perilesional T2-flair; non-enhancing disease is at most risk for subsequent progression. Furthermore, it has been demonstrated that a subset of gliomas may also exhibit a more aggressive clinical course after surgery and have radiographic recurrence prior to initiating adjuvant chemotherapy and radiation. These HGGs, termed rapid early progressors (REP) are associated with decreased progression free and overall survival [5]. Currently, there is no standard pre-operative model that facilitates REP prediction.

An improved understanding of the extent of disease at the time of diagnosis would be clinically informative. Gaining the ability to predict regions of tumor recurrence would facilitate treatment planning. Artificial intelligence (AI) and machine learning (ML) are increasingly utilized tools that facilitate diagnostic and therapeutic advances in medicine [6]. Previously, these tools have been applied in the discrimination between tumor recurrence and treatment-related changes in HGGs [7, 8]. As a result, interest in developing AI and ML models which integrate conventional and advanced radiographic imaging features into a recurrence prediction model has grown. Such a model would enable a highly personalized approach to managing HGGs.

In this review, we evaluated and synthesized the existing literature reporting on AI and ML approaches to predicting tumor recurrence in HGGs. Additionally, we discuss future applications and develop unanswered clinical questions.

## Methods

### Study design and inclusion criteria

We conducted a systematic review in accordance with the Preferred Reporting Items in Systematic Reviews and Meta-Analyses (PRISMA) guidelines [9]. The study aimed to examine the existing literature reporting on tumor recurrence prediction in HGGs (defined as World Health Organization (WHO) grades III and IV) with the use of AI or ML techniques, including neural networks (NN). Studies were included if they met the following criteria: (1) Histopathologic diagnosis of WHO grade III or IV glioma (2) imaging subject to AI/ML/NN modeling (3) quantitative or qualitative analysis on the performance of the model with respect to detecting tumor recurrence. The studies included herein span two WHO grading editions and therefore the definition of HGG adheres to the updated 4th edition (2016) WHO grading scheme in articles published prior to the updated 5th edition in 2021 [10, 11].

### Search strategy

Three primary databases were searched for relevant literature: PubMed, ScienceDirect, and Web of Science. Each database was systematically searched from their inception through March 2025. The following keywords were included in the search: “High-grade-glioma”, “Glioblastoma”, “WHO grade III glioma”, “WHO grade IV glioma”, “Anaplastic Astrocytoma”, “Gliosarcoma” “Artificial Intelligence”, “Machine Learning”, “Neural Network”, “recurrence”, “prediction.” Medical Subject Headings (MeSH) terms in PubMed were also utilized. The reference section of selected articles was reviewed for relevant publications.

### Study selection

Non-English language texts were excluded from analysis. Meta-analyses, reviews, conference abstracts, grey literature, book chapters, editorials, and case reports were also excluded from analysis. Studies which reported data on WHO grade I and II, lacked sufficient data on outcomes, and did not analyze image-based recurrence prediction were excluded. The literature search was conducted in parallel by two independent authors (S.J) and (T.K), disagreements over discrepancies in the final article inclusion list were resolved by group consensus.

### Data extraction

The following data was extracted from selected articles and uploaded into a pre-designed excel sheet: Author names, study design, number of patients, WHO grade, imaging modalities, recurrence criteria, performance metrics (sensitivity, specificity, area under the curve (AUC), accuracy), and index treatment. Two authors extracted data independently (T.K) and (S.J), with discrepancies resolved by group consensus.

### Quality assessment

The Joanna Briggs Institute (JBI) critical appraisal tool for cohort studies was used to evaluate potential manuscripts for inclusion into the final analysis. Manuscripts were included if they scored low to moderate risk of bias (≥ 60%).

### Statistical analysis

Continuous and categorical variables were reported as medians (range) or event rate (%) where appropriate. A pooled analysis of sensitivity, specificity, and accuracy was conducted with results graphically displayed as forest plots. A random effects model was selected with point estimates obtained using inverse variance weighting. To account for between study heterogeneity, particularly in the case of a limited number of studies we applied the Hartung-Knapp adjustment to the standard error of the overall effect estimates [12]. Point estimates for sensitivity, specificity, and accuracy were derived from the highest performing models in each study, which was the external validation (“test cohort”) for each respective study. The event rate for sensitivity values represents the proportion of true positive rates, which was backcalculated using the formula: *true positive = (sensitivity X (true positive +false negative)).* Similarly, the event rate for the specificity values represents the true negative rates, which were backcalculated using the formula: *true negative= (specificity X (true negative + false positive)).* The I^2^ statistic was calculated to capture inter-study heterogeneity with an I^2^ value of < 30%, 30–60%, and > 60% defined as low, moderate, and high heterogeneity, respectively. To investigate and reduce between-study heterogeneity, leave-one-out sensitivity analyses were performed, sequentially removing individual studies until heterogeneity was minimized, ideally approaching 0%. Analysis was performed with GraphPad Prism version 10.3.1 and the *meta*,* RTSA*, and *metafor* packages in R programming environment version 4.2.2.

## Results

### Study characteristics

**Fig. 1:**
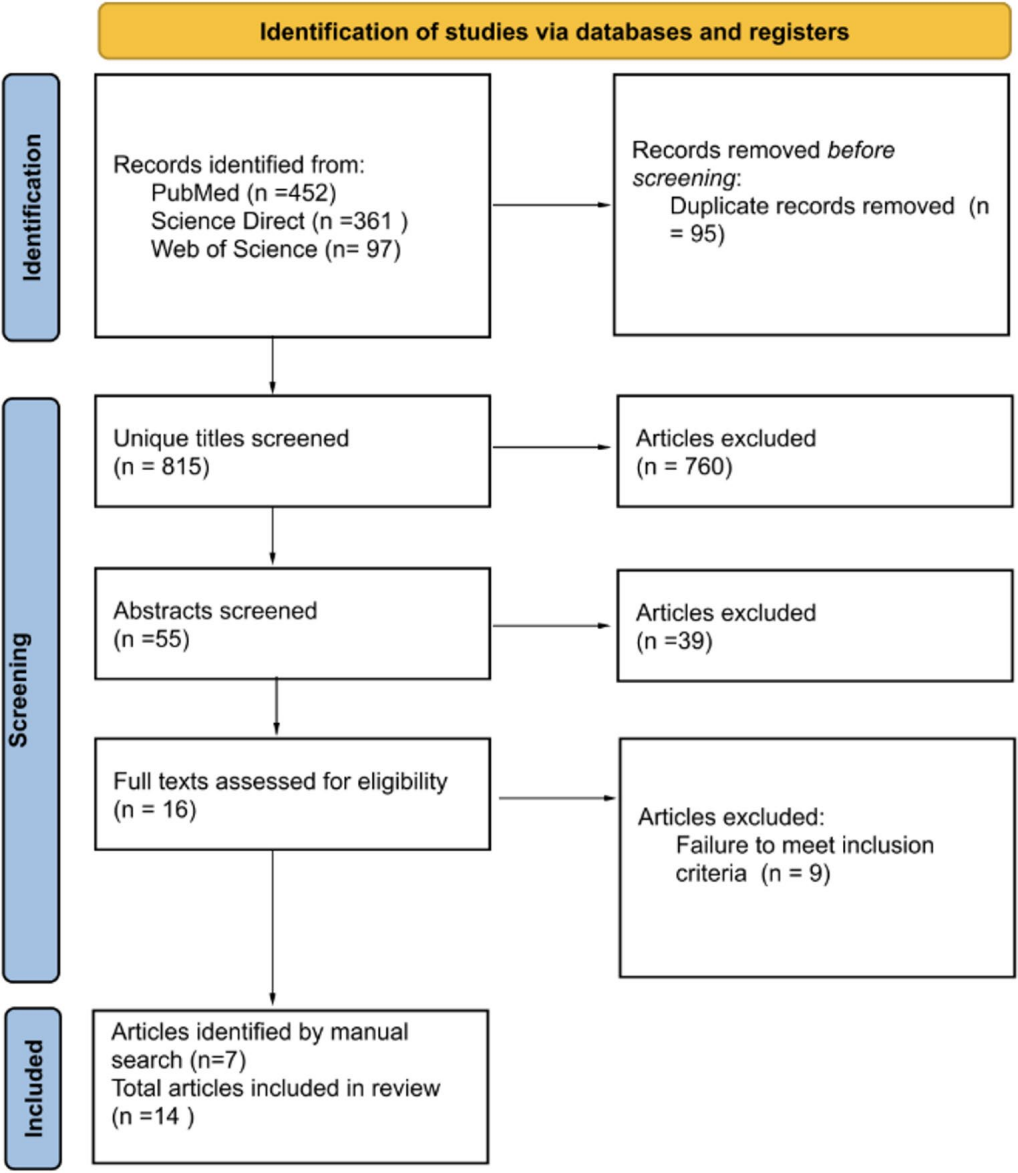
PRISMA flowchart describing study selection process

A PRISMA diagram detailing the literature search selection process is outlined in Fig. 1. The initial literature search yielded 910 articles, which was reduced to 815 following the removal of duplicate articles. Following a title screen of these 815 articles, 760 were excluded due to a lack of keyword concordance. The remaining 55 articles were screened based on their abstracts, with 39 articles undergoing subsequent exclusion. Finally, 16 articles underwent full text review with 9 excluded resulting in a total of 7 articles identified from the search process; a manual search was performed based on article references with 7 additional articles identified for a total of 14 articles eligible for review (Fig. 1). Overall, the studies included were either retrospective (13/14, 92.9%) or prospective (1/14, 7.1%) (Table 1). In total, 1,540 patients were included with (1,530, 99.3%) Grade IV and (10, 0.7%) Grade III histology. The index procedure in each study consisted of either gross total or subtotal resection followed by Stupp protocol (13/14, 92.9%) [2]. There was a total of 13 unique algorithms used with Random Forest (RF) being the most frequent (Table 2). More than half (8/14, 57.1%) utilized the Response-Assessment in Neuro-Oncology (RANO) for assessment of recurrence (Table 1).

**Table 1:** Baseline study characteristics

Author/Year	Study Design	Number of Patients	WHO Grade (%)	Index Treatment	Recurrence criteria
Zhu et al. [24] (2025)	Retrospective	71	III 7 (9.9) IV 64 (90.1)	GTR/NTR+STUPP	RANO criteria
Kwak et al. [25] (2024)	Retrospective	143	IV (100)	GTR+STUPP	Histopathological
Pignotti et al. [26] (2024)	Retrospective	248	IV (100)	GTR + RT or Carmustine Wafers	NR
Yoon et al. [20] (2024)	Retrospective	179	IV (100)	GTR+STUPP	Measurable enhancing lesion within non-enhancing T2-hyperintense area around surgical cavity
Rivera et al. [18] (2024)	Prospective	16	III 3 (18.7) IV 13 (81.3)	GTR+STUPP	RANO criteria
Cepeda et al. [14] (2024)	Retrospective	55	IV (100)	GTR+STUPP	Modified RANO criteria
Heo et al. [27] (2023)	Retrospective	212	IV (100)	GTR/STR+STUPP	RANO criteria
Du et al. [28] (2023)	Retrospective	134	IV (100)	GTR+STUPP	RANO criteria
Chougule [17] (2022)	Retrospective	29	IV (100)	GTR+STUPP	> 25% increase in sum of products of perpendicular diameters of enhancing lesions, any new enhancing lesion beyond resection margins, progressive increase in rCBV on interval imaging
Shim et al. [29] (2021)	Retrospective	192	IV (100)	GTR+STUPP	RANO criteria
Yan et al. [15] (2020)	Retrospective	57	IV (100)	GTR/STR+STUPP	RANO criteria
Kim et al. [30] (2019)	Retrospective	83	IV (100)	GTR/STR/+STUPP	RANO criteria
Rathore et al. [1] (2018)	Retrospective	90	IV (100)	GTR+STUPP	Histology proven recurrence in pre-defined ROI
Akbari et al. [13] (2018)	Retrospective	31	IV (100)	GTR+STUPP	Contrast enhancing areas identified by a neuro-radiologist on immediate post-operative MRI

*GBM* Glioblastoma, *WT* Wild-type, *NR* not reported, *GTR* gross total resection, *STUPP* 60 Gy EBRT in 30 fractions with concurrent Temozolomide chemotherapy, *HGG* high-grade glioma, *RANO* response assessment criteria in neuro-oncology, *ROI* Region of interest, *rCBV* relative cerebral blood volume

**Table 2:** Technical details and key performance metrics

Author/Year	Input Sequences	AI/ML Algorithm	Validation	Accuracy	Sensitivity	Specificity	Area Under Curve (AUC)
Zhu et al. [24] (2025)	T1, T1CE, T2-FLAIR	Gradient boosting	Internal	NR	NR	NR	1.0
Kwak et al. [25] (2024)	T1, T1CE, T2-FLAIR, ADC, DTI	CNN	Leave-one-out	NR	0.80	0.88	NR
Pignotti et al. [26] (2024)	T1, T1CE, T2	XGBoost	External	NR	0.62	0.80	0.72
Yoon et al. [20] (2024)	T1, T1CE, T2-FLAIR, DCE	nnU-Net	NR	NR	0.80	0.50	NR
Rivera et al. [18] (2024)	WB-MRS, T1, T1CE, T2-FLAIR	Naive Bayes, Logistic regression, Decision Tree, RF, Gradient boosting, ANN	5-fold	0.70	NR	NR	0.86
Cepeda et al. [14] (2024)	T1, T1CE, T2, T2-FLAIR, ADC	CatBoost, XGBoost, RF, LightGBM	voxelwise and regionwise	0.88	NR	NR	0.81
Heo et al. [27](2023)	T1, T2-FLAIR, DSC	nn-Unet	Internal	0.54	0.80	0.48	0.52
Du et al. [28](2023)	T1, T1CE, T2-FLAIR, DSC	Decision Tree	External	NR	0.81	0.81	0.81
Chougule et al. [17] (2022)	T1CE, FLAIR, ADC	RF	3-fold	0.71	0.66	0.74	NR
Shim et al. [29] (2021)	T1, T2-FLAIR, DSC	Multilayer perceptron	5-fold	NR	NR	NR	0.97
Yan et al. [15] (2020)	MPRAGE, T2, T2-FLAIR, DTI, DSC[1],H MRS	nntool	cross entropy	0.92	0.80	0.97	NR
Kim et al. [30] (2019)	T1, T1CE, T2-FLAIR, DWI, DSC	Generalized Linear Model	10-fold	NR	0.80	0.63	0.67
Rathore et al. [1] (2018)	T1, T1CE, T2, T2-FLAIR, DTI, DSC-MRI	SVM	leave-one-out	0.89	0.97	0.76	0.91
Akbari et al. [13] (2016)	T1, T2, T2-FLAIR, DTI, DSC-MRI	SVM	Leave-one-out	0.87	0.93	0.88	0.80

*T1CE* contrast enhanced T1 MRI, *FLAIR* fluid attenuated inversion recovery, *ADC* apparent diffusion coefficient, *DTI* diffusion tensor imaging, *DSC* dynamic susceptibility contrast, *CNN* convolutional neural network, *XGBoost* extreme gradient boost, *nnU-net*: neural network, *RF* random forest, *CatBoost* categorical boost, *nntool* neural network tool, *SVM* support vector machine

The following imaging modalities were utilized as input sequences into the models: T2-FLAIR (13, 92.9%), T1W (12, 85.7%), T1CE (9, 64.3%), DSC-MRI (7, 50.0%), DTI (4, 28.6%), ADC (2, 14.3%), DCE-MRI (1, 7.1%), MPRAGE (1, 7.1%), WB-MRS (1, 7.1%), ^1^H-MRS (1, 7.1%) (Table 2). A total of 8 unique validation strategies were utilized with leave-one-out cross validation the most frequent (Table 2). The sensitivity, specificity, accuracy, and area under the curve ranged from 0.62 to 0.97, 0.48–0.97, 0.54–0.92, and 0.52–01.0 respectively (Table 2).

### Performance metrics

**Fig. 2:**
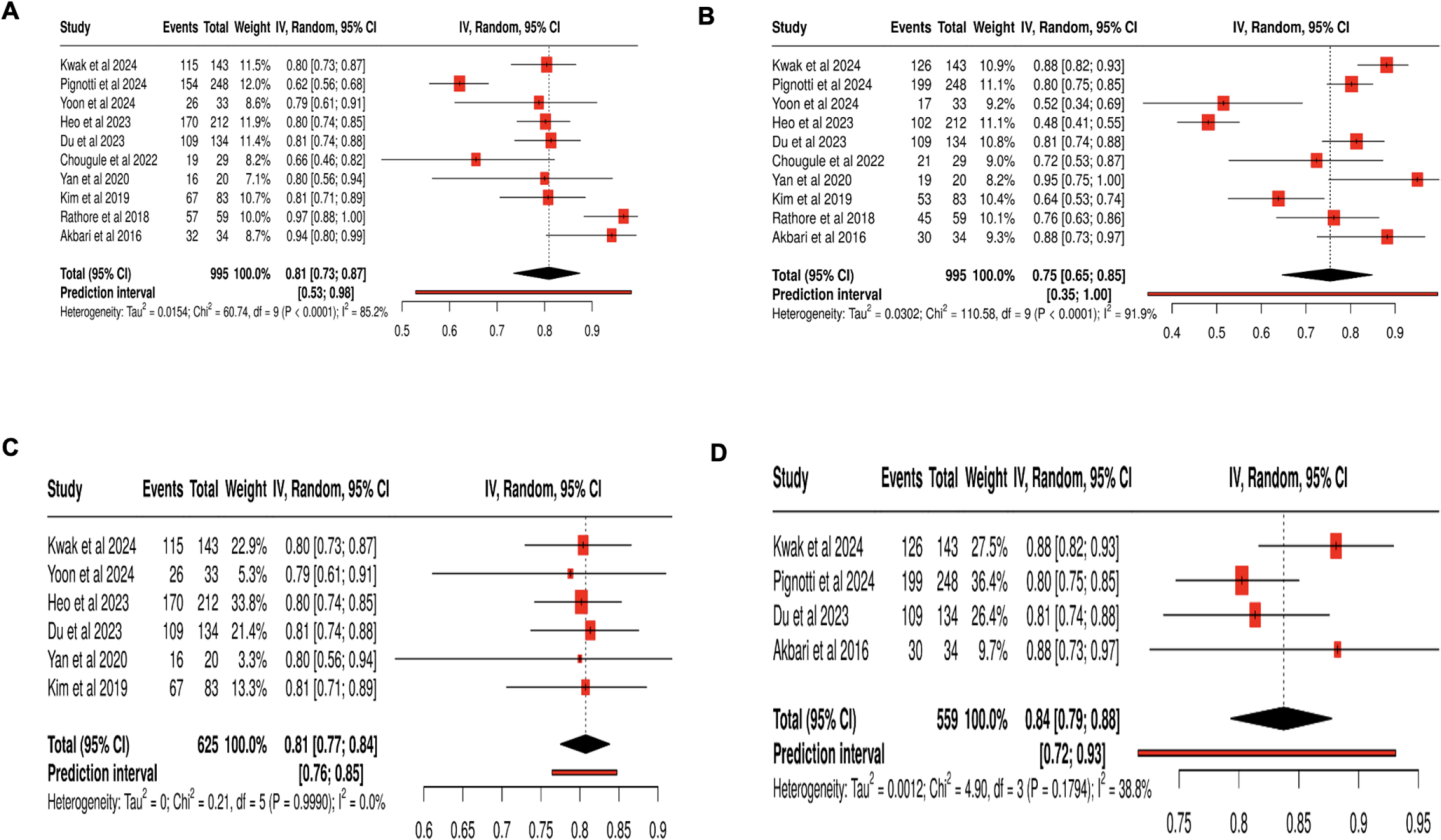
Pooled analysis of key performance metrics. Sensitivity (**A**) Specificity (**B**) with corresponding sensitivity analyses (**C**) (**D**)

**Fig. 3:**
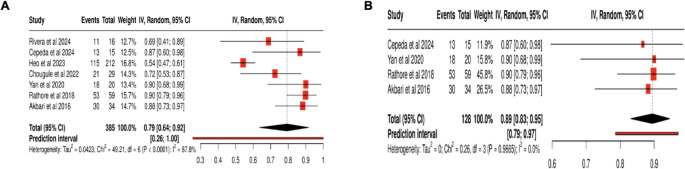
Pooled analysis of Accuracy (**A**) with corresponding sensitivity analysis (**B**)

The pooled sensitivity, specificity, and accuracy of the models were 81% (95% CI: 73–87; I² = 85.2%), 75% (95% CI: 65–85; I² = 91.9%), and 79% (95% CI: 64–92; I² = 87.8%), respectively (Figs. 2A-B and 3A). Following sensitivity analyses, the corresponding estimates were 81% (95% CI: 77–84; I² = 0.0%), 84% (95% CI: 79–88; I² = 38.8%), and 89% (95% CI: 83–95; I² = 0.0%), respectively (Figs. 2C and D and 3B).

## Discussion

### An overview of technical parameters

The availability of medical imaging for research has expanded over the past decade. Additionally, the integration of AI and ML applications has become increasingly recognized. Current research has focused largely on the augmentation of contemporary imaging capabilities at the most basic level utilizing T1-weighted (T1-W), T2-W, and T2-FLAIR (fluid attenuated inversion recovery) inputs for AI/ML processes [1, 6–8, 10–18]. These imaging modalities facilitate learning and modeling areas of contrast enhancement and differentiating regions of edema from cellular infiltration; more advanced imaging modalities have been utilized to increase the sophistication of these models. In one of the earliest reports, the use of diffusion tractography Imaging (DTI) and diffusion susceptibility contrast imaging (DSC-MRI) were added to facilitate interpretation beyond the limits of T1-W, T2-W, and T2-FLAIR scans [6]. With the advent of DTI, researchers were able to identify white matter tracts and their relationship to the adjacent tumor. For the purpose of AI/ML modeling, Akbari et al. defined several key imaging features including radial diffusivity (RAD), fractional anisotropy, axial diffusivity (AX), and trace (TR) which could be extracted and leveraged to determine regions of probable tumor infiltration [13]. The use of DSC-MRI in the work of Akbari et al. represented one of the earliest applications of functional imaging for AI/ML modeling for central nervous system (CNS) tumors [13]. Key metrics derived from this imaging modality are relative cerebral blood flow (rCBF), relative cerebral blood volume (rCBV), and mean transit time (MTT) [13]. Through a principal component analysis, the highest contributing elements of DSC-MRI are used to synthesize the overall cerebral perfusion dynamics, which are altered in the presence of tumor infiltration [13]. Following the work of Akbari and Rathore et al., the first instance of ^1^H MR spectroscopy data acquisition for AI/ML in CNS tumor detection was demonstrated by Yan et al. [15]. MR spectroscopy enables the quantification of metabolites, most notably N-acetyl aspartate (NAA), and Choline (Cho), further operationalized as a ratio of Cho/NAA to estimate cellular turnover within a target radiographic region [15]. This reinforced the utilization of dynamic imaging parameters to augment the robustness of AI/ML processes. More recently, a manuscript by Rivera et al., whole-brain MR spectroscopy (WB-MRS) was utilized to predict regions of glioblastoma infiltration [18].

Regardless of which imaging modalities are selected for study, the basic principles and processes in place for extracting and applying radiographic features are generally consistent. The overall process can be defined by the following steps: image registration, segmentation, and radiographic feature extraction. The initial step in developing an AI/ML model involves the process of image registration, a method of aligning multiple images into a cohesive coordinate system. Following this, the user must define “regions of interest” (ROIs) to facilitate segmentation. The process of segmentation is often accomplished by utilizing commercial software, for example “Glioma Image Segmentation and Registration” (GLISTR) [1, 6]. Leveraging the newly defined ROIs following image segmentation, the process of feature extraction takes place which allows for the identification of unique radiographic signatures at the voxel level. Finally, an ML pattern classifier is utilized to correlate radiographic features with real world examples of the pathology in question; common choices for such pattern classifiers include support vector machines (SVM) and Random Forest classifiers (RF).

### Contemporary models

Akbari et al. were one of the first to attempt the application of an AI/ML model to the radiographic prediction of future glioblastoma recurrence [13]. In that study, images were segmented with the GLISTR protocol with three distinct image labels defined: enhancing lesion, non-enhancing lesion, and peritumoral edema [13]. The peritumoral region was further sub-classified as “near” and “far” with near regions characterized as immediately adjacent to the enhancing lesion [13]. The method of labeling in this study was based on an inherent assumption of increased tumor infiltration near the enhancing component of the lesion. An SVM classifier was subsequently trained on voxels from these pre-labeled regions and applied to a testing cohort to derive a probabilistic tumor infiltration map [13]. Infiltration maps were compared to regions of recurrence on post-resection imaging with promising concordance [13]. Following their work, Rathore et al. redemonstrated the ability to delineate areas of tumor infiltration within the peritumoral space and predict regions of early glioblastoma recurrence [1]. Rathore et al. enhanced their model training with the additional use of quantitative radiomic features which included: signal intensity, distance of voxels, local mean and median of voxels, local voxel contrast and entropy, and perfusion temporal dynamics [1]. Once again, probabilistic tumor infiltration maps were compared to post-resection images with strong concordance [1].

The ability to discern regions of densely infiltrated tumor within the peritumoral space was further confirmed in 2020 by Yan et al., albeit with the use of neural networks which are currently the state of the art in terms of performance [15]. Interestingly in their work, the authors registered post-surgical images demonstrating radiographic progression with pre-surgical images and trained a neural network tool to predict progression in an external validation cohort with 92.4% accuracy [15]. In 2023, Cepeda et al. were the first group to validate their recurrence prediction model for grade IV gliomas using multi-institutional data [14]. Furthermore, the 3-dimensional graphical representations of future tumor recurrence exceeded the work of their predecessors. A group from the University of Miami was the first to employ a novel whole-brain magnetic resonance spectroscopy (WB-MRS) based AI model that was developed and applied to the prediction of radiographic glioblastoma recurrence [18]. The authors were able to successfully differentiate areas of tumor recurrence based on five key metabolites: Cho, NAA, Cr, Glx, and myo-inositol [18].

Most recently, Yoon et al. trained a publicly available nnU-Net, a neural network model which leveraged conventional and DCE-MRI to detect differential vessel “leakiness” across areas of recurrence and non-recurrence [20, 21]. This study was also one of the first to directly compare model sensitivities between models relying on conventional MR images and advanced MR imaging with DCE-MRI [13]. The results of this analysis demonstrated superior performance of the advanced imaging cohort (80%) versus the conventional imaging group (40%) (*p* = 0.03) with respect to recurrence model sensitivity [20].

### Clinical implications

Recurrence in HGGs is almost inevitable; consequently, persistent debate surrounding the extent of surgical and clinical management exists. Standard surgical planning in patients with HGGs focuses on targeting contrast enhancing regions on post-contrast T1-W MRI [1, 6]. Unfortunately, it is well understood that residual regions of infiltrating tumor, which may not necessarily demonstrate enhancement on post-contrast MRI, may be left behind after surgical excision. This is supported by the fact that most recurrences are noted around the contrast enhancing tumor margin [13]. Indeed, Shah et al. demonstrated a survival benefit following resection of the surrounding FLAIR signal compared to exclusively resecting the contrast enhancing region [19]. While radiation therapy to an expanded clinical target volume around the tumor is typically employed following surgical excision, the dose is applied uniformly, and without respect to regions of differential cell density [14, 15]. Therefore, more precise surgical and radiosurgery planning could be achieved with an improved understanding of the spatial extent of the non-enhancing infiltrative tumor component in glioblastoma [22, 23].

The utilization of advanced methodologies like AI and ML have demonstrated the ability to discriminate tumor infiltration in the setting of HGG, and more importantly predict regions of recurrence with reasonable accuracy [1, 6–8, 10–13]. Ultimately, this could facilitate greater extent of resection, increasingly individualized surgery, and in some cases supra-maximal resection. Improvement in the extent of resection is critically important to improvement in progression-free survival (PFS), which in the setting of HGG is a surrogate for overall survival [1]. Regardless, promoting improved PFS and delaying progression enables the introduction and escalation of alternative therapies or enrollment in clinical trials. Furthermore, radiation dose-escalation strategies in regions of high tumor infiltration may be applied for improved local tumor control. However, caution must be exercised when planning a dose-escalation strategy as the therapeutic index can be narrow. Therefore, precisely identifying regions of the highest tumor cell to healthy parenchyma is paramount for reducing radiation toxicity.

Overall, the studies reviewed herein demonstrated that there exists a set of radiographic features consistently associated with regions of tumor infiltration imperceptible to human recognition. As it stands, clinical decision-making regarding HGG often occurs after significant progression has occurred, therefore decision making can be limited with respect to time and eligibility for alternative therapy. Beyond the scope of HGGs, many of the fundamental processes and findings demonstrated by these studies may apply to other tumors both CNS and non-CNS in origin.

### Technical limitations

One of the most critical limitations seen in these studies is the absence of histopathological ground truth from the infiltrated, peritumoral areas [6]. Additionally, in models employing the ROI approach, arbitrary designations based on the assumed distribution of tumor cells near and far from the enhancing core may be fundamentally flawed. Development and training of a robust AI/ML model is contingent on the extraction of highly granular data, which is related to the number of interval MRI scans. In real-world application, patients may only obtain imaging every three or six months which limits model training. Another concern across the studies was the degree to which the contrast enhancing regions were surgically excised. The contrast enhancing tumor was not uniformly excised across each subject in every study, therefore residual enhancing tumor may have interfered with analysis in cases of “sub-total’ resection. Many of the studies have been limited to single institutions, limiting their generalizability. The retrospective nature of these studies subjects them to potential selection bias. Finally, there exists inconsistency by which tumor recurrence is defined across these cohorts. It is imperative that investigators in this space adopt standardized criteria by which they measure recurrence; a widely accepted example of this is RANO criteria.

## Conclusions

Over the past decade there has been an effort to define regions of tumor recurrence following surgical excision in HGGs using AI and ML. While the literature is sparse, there is preliminary evidence to suggest that predicting post-operative tumor recurrence is possible using both standard imaging sequences as well as advanced modalities. Refining these AI/ML models by incorporating histopathologic analysis will facilitate model robustness. In addition to revolutionizing the surgical outcomes of HGGs, this technology may be applied to other tumors inside and outside of the central nervous system.

## Data Availability

No datasets were generated or analyzed during the current study.
